# Development of Multiplexed Bead-Based Immunoassays for the Detection of Early Stage Ovarian Cancer Using a Combination of Serum Biomarkers

**DOI:** 10.1371/journal.pone.0044960

**Published:** 2012-09-10

**Authors:** Yong-Wan Kim, Su Mi Bae, Hyunsun Lim, Yoon Ji Kim, Woong Shick Ahn

**Affiliations:** 1 Catholic Research Institutes of Medical Science, College of Medicine, The Catholic University of Korea, Seoul, Korea; 2 Gangnam Severance Hospital Biomedical Research Center, Yonsei University College of Medicine, Seoul, Korea; 3 Department of Obstetrics and Gynecology, College of Medicine, The Catholic University of Korea, Seoul, Korea; University Clinic of Navarra, Spain

## Abstract

CA125 as a biomarker of ovarian cancer is ineffective for the general population. The aim of this study was to evaluate multiplexed bead-based immunoassay of multiple ovarian cancer-associated biomarkers such as transthyretin and apolipoprotein A1, together with CA125, to improve the identification and evaluation of prognosis of ovarian cancer. We measured the serum levels of CA125, transthyretin, and apolipoprotein A1 from the serum of 61 healthy individuals, 84 patients with benign ovarian disease, and 118 patients with ovarian cancer using a multiplex liquid assay system, Luminex 100. The results were then analyzed according to healthy and/or benign versus ovarian cancer subjects. When CA125 was combined with the other biomarkers, the overall sensitivity and specificity were significantly improved in the ROC curve, which showed 95% and 97% sensitivity and specificity, respectively. At 95% specificity for all stages the sensitivity increased to 95.5% compared to 67% for CA125 alone. For stage I+II, the sensitivity increased from 30% for CA125 alone to 93.9%. For stage III+IV, the corresponding values were 96.5% and 91.6%, respectively. Also, the three biomarkers were sufficient for maximum separation between noncancer (healthy plus benign group) and stage I+II or all stages (I−IV) of disease. The new combination of transthyretin, and apolipoprotein A1 with CA125 improved both the sensitivity and the specificity of ovarian cancer diagnosis compared with those of individual biomarkers. These findings suggest the benefit of the combination of these markers for the diagnosis of ovarian cancer.

## Introduction

Ovarian cancer has a higher fatality-to-case ratio than any other gynecologic malignancy, since it tends to be complex by symptoms and misdiagnosed than other diseases, which results in the vast majority of patients with ovarian cancer being diagnosed in advanced metastatic stages (stage III/IV) [1]–[3]. The 5-year survival rate of patients with early stage cancer ranges from 50–95%, but only approximately 20% of all reported cases are caught in the early stages; the 5-year survival rate is approximately 11% when detected in the advanced stages (III/IV) [4]–[6]. Therefore, many efforts have been focused on the identification of diagnostic biomarkers for early detection of ovarian cancer [7], [8]. A robust detection method based on molecular profiles for ovarian cancer has not yet been established because the disease exhibits metabolic changes due to the presence of the tumor and potential genetic variations that affect blood chemistry during the course of tumor progression [9].

The cancer antigen 125 (CA125) assay is the most used clinical biomarker for ovarian cancer [10]. However, CA125 has proven to be a poor diagnostic tumor biomarker because it lacks specificity and sensitivity for early ovarian cancer (only 23% in stage I ovarian cancer, in contrast to more than 80% in advanced ovarian cancer) [11]. It is elevated above reference levels in only 50% of clinically detectable early stage disease, and is not infrequently elevated in patients with benign ovarian diseases [12], [13]. In addition, CA125 levels are falsely elevated in pregnant women and women with detectable intraperitoneal pathologies [14]–[16]. Therefore, attempts have been made to combine or replace CA125 with other markers, and investigators have evaluated the ability of some established markers to improve the identification and prognosis of ovarian cancer [12], [17], [18], thus indicating that the addition of one or several markers to CA125 would improve diagnostic and prognostic performance if sensitivity was improved without a loss in specificity. However, because the measurement of serum concentration of each putative biomarker with individual ELISAs requires considerable time, cost, and sample volumes, new methods or technologies for multiplexing must be developed.

The Luminex bead-based system is a automated high-throughput assay platform that provides multiplexing in a solution phase, resulting in it being particularly flexible and nondestructive for protein analysis. The use of detection antibodies labeled with biotin and streptavidin-R-phycoerythrin allows quantification of antigen-antibody reactions that occur on the microsphere surface through the measurement of the relative fluorescence intensity. Therefore, the system is capable of measuring up to 100 analytes simultaneously in a small sample volume (less than 50 μl), indicating multivariate methods that use a panel of biomarkers to predict specific clinical end points of interest.

In this study, we attempted to measure three serum biomarkers of ovarian cancer, CA125, transthyretin, and apolipoprotein A1, using a multiplex bead-based immunoassay system, and evaluated the combined effect of the three biomarkers for the diagnosis of ovarian cancer compared with those of the individual markers alone.

## Materials and Methods

### Ethics statement

All patients involved in the study had signed a declaration of consent stating that the patients specimens may be used for scientific intentions. Specimens were obtained from the patients in the Department of Obstetrics and Gynecology in concordance with procedures approved by the Institutional Review Board of The Catholic University of Korea (07BR212).

### Patients and samples

All patients were enrolled at St. Mary’s Hospital of Catholic Medical School during the period from January 2001 to July 2007, according to the procedures approved by the Institutional Review Board of The Catholic University of Korea. This study was based on analyses of serum collected from 118 patients with ovarian cancer, 84 with benign disease, and 61 healthy females. Patient serum samples were collected before surgery, and then incubated for 30 min at room temperature, followed by centrifugation at 3,000 rpm for separation. The serum was stored at −70°C until used in experiments; frequent freezing and thawing were avoided. The stages and grades of tumors from the ovarian cancer patients were assigned according to the guidelines provided by the International Federation of Gynecology and Obstetrics (FIGO), and the enrolled groups were then divided according to age.

### Conjugation of primary antibodies with microspheres

Three different kinds of microspheres (1×10^6^ microspheres for each antibody, Biosource, Camarillo, CA) were prepared in each tube, and were then resuspended well by vortexing and sonication, followed by centrifugation for 2 min at 8,000 rpm. Supernatants were discarded, and the pellets were saved and washed once with 100 μl saline. Eighty μl of 100 mM monobasic sodium phosphate (pH 6.2, Sigma-Aldrich, St. Louis, MO), 10 μl of 50 mM Sulfo-NHS (Pierce Biotechnology, Rockford, IL) and 10 μl of 50 mM EDC (Pierce Biotechnology) were added, and the solution was then incubated for 20 min at room temperature. After centrifugation (8,000 rpm, 2 min), the pellets were saved and washed twice with 250 μl of 50 mM MES (pH 5.0, Sigma-Aldrich). After the removal of the supernatant, 500 μl of MES was added to each tube including different microspheres. Following the addition of 0.5 μg of each antibody [anti-CA125 (Fitzgerald Industries International, Inc., Concord, MA), anti-transthyretin (Abcam), and anti-apolipoprotein A1 (Fizgerald Industries International, Inc.)] in each tube, the tubes were incubated for 2 h on a shaker, which was protected from the light. After the incubation, antibody-bound microspheres were pelleted by centrifugation for 2 min at 8,000 rpm, and 500 μl of 1% BSA buffer was then added. After additional incubation for 30 min at room temperature, the microspheres were washed twice with 1% BSA buffer and then stored at 4°C under protection from light.

### Labeling biotins on the secondary antibodies

For labeling biotins on the secondary antibodies, a biotin labeling kit (Alpha Diagnostics International Inc., San Antonio, TX) was used according to the protocol of the manufacturer. Briefly, biotin was added at a ratio of 1∶10 (biotin:antibody). After incubation for 1 h at room temperature under protection from light, dialysis was performed with phosphate-buffered saline (PBS).

### Analysis of samples by multiplex liquid array system, Luminex 100

The serum from healthy individuals and ovarian cancer patients were diluted to 1∶100 in a buffer including 1% BSA (Sigma-Aldrich) and 0.05% Tween 20 (Sigma-Adrich). Fifty μl of each diluted serum were plated on a 1.2 μm filter plate (96 well), to which 2,500 beads of each antibody-bound microsphere were added in 50 μl. After incubation for 2 h at room temperature under protection from light, they were washed twice with PBS buffer including 0.05% Tween 20. One hundred μl of 0.4 μg of streptavidin-R-phycoerythrin (Sigma-Aldrich) was added to each well, and plates were then incubated for 30 min, followed by two washes with PBS containing 0.05% Tween 20. The identification of antibody-bound microspheres and the screening of antigen-antibody-bound microspheres were carried out by using Luminex 100 (Luminex Corp, Houston, TX) according to the protocol of the manufacturer. Ranges of the concentrations of each antigen for standard curves were 10–250 U/ml for CA125, 0.1–100 μg/ml for transthyretin, and 0.5–50 ng/ml for apolipoprotein A1. The data were analyzed by the BeadView program (Upstate, Charlottesville, VA).

### Statistical analysis

The analysis of variance (ANOVA) test was used to assess the statistical significance of differences between the healthy individuals and ovarian cancer patients. SigmaPlot (v12.0, Systat, Chicago, IL) and SAS (v9.1, SAS Institute, Cary, N.C., USA) was used for statistical analysis to determine the sensitivity, specificity, and the receiver operator characteristic (ROC) curve (REMARK in File S1).

### REMARK criteria

A description of the fulfilment of Reporting Recommendations for Tumor Marker Prognostic Studies (REMARK) [19] criteria for biomarker studies is provided in File S1.

## Results

### Serum levels of ovarian tumor markers in healthy control, benign, and ovarian cancer groups

**Figure 1:**
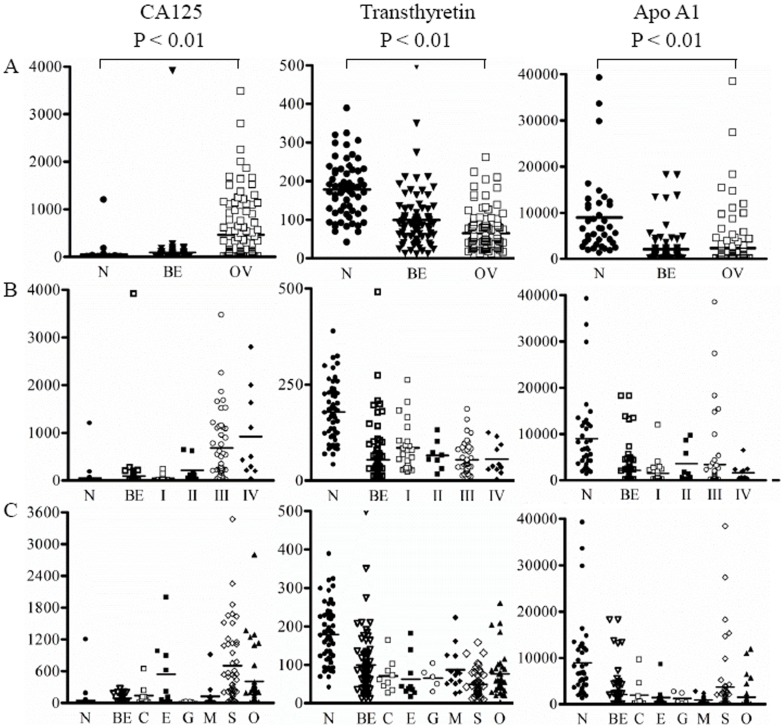
Scatter plots of concentrations of CA125, transthyretin, and apolipoprotein A1. (A) healthy controls, benign ovarian disease and ovarian cancer patients (B) tumor stages in ovarian cancer patients (C) different histological subtypes in ovarian cancer patients. P value over a group denotes statistical significance of differences between each group and healthy controls. Each ovarian cancer subjects was compared with healthy controls. N, healthy controls; BE, benign ovarian disease; C, clear cell; E, endometrioid; G, granulosa cell; M, mucinous; S, serous; O, other.

The characteristics of patients and serum levels of ovarian cancer markers are shown in Table 1. Concentration of serum biomarkers such as CA125, transthyretin, and apolipoprotein A1 in serum from healthy individuals, benign patients, and ovarian cancer patients was simultaneously measured by a multiplex liquid array system using microbeads coated with capture antibodies and biotin-labeled antibodies against each of the tumor markers and streptavidin-R-phycoerythrin. The serum levels of CA125 was significantly higher in ovarian cancer patients than that in healthy individuals and benign patients, while the levels of transthyretin and apolipoprotein A1 were lower in ovarian cancer patients (Fig. 1A). First, we compared the serum levels of these three tumor markers according to the tumor stages (Fig. 1B). The serum levels of CA125 were gradually elevated with tumor stage. Also, both transthyretin and apolipoprotein A1 were significantly increased in healthy individuals. Next, we attempted to compare the serum levels of three tumor markers according to histologic types of ovarian cancer (Fig. 1C). The serum level of CA125 was the highest in serous type compared with those in the other types.

**Table 1:** Concentration of serum markers with clinicopathological findings in ovarian cancer patients.

Characteristics		Age (years)	No. of group	CA125 (U/ml)	Transthyretin (μg/ml)	Apolipo A1 (μg/ml)
		Median Mean				
Healthy normal (control)		43 43.5	61 (100%)	13.8±4.1*	173.5±50.9	6875.0±4044.1
Benign patients		36 37.9	84 (100%)	41.6±30.1	90.6±39.7	1369.6±1140.4
Ovarian cancer patients		54 52.6	118 (100%)	285.8±397.6	53.1±34.0	815.4±1947.3
FIGO Stage	I	52.6 47.1	24 (26.1%)	30.2±24.5	73.8±37.8	942.2±737.1
	II	49 45.1	8 (8.7%)	179.4±66.8	62.9±23.8	3081.7±2162.3
	III	62 57.1	49 (53.2%)	567.5±363.2	46.8±24.8	1340.5±1093.3
	IV	56 54.3	11 (11.9%)	798.9±722.3	46.1±33.5	1077.0±860.6
Histological subtype	Serous	58 56.4	46 (38.9%)	582.0±457.2	43.7±23.5	1625.6±1263.5
	Mucinous	54 46.8	14 (11.8%)	61.0±70.3	78.4±27.1	785.7±547.4
	Clear cell	47.5 44.6	9 (7.6%)	84.9±78.1	64.1±23.6	1092.8±592.7
	Endometrioid	42 45.0	10 (8.5%)	411.9±409.4	41.7±37.6	566.5±467.3
	Granulosa cell	36.5 38.5	5 (4.2%)	15.9±4.1	65.3±15.0	1123.3±1062.5
	Other	54 54.8	34 (28.8%)	292.8±306.2	60.2±26.2	779.6±522.6

* Values are presented as Mean.

### Comparison of the sensitivity and specificity between three tumor markers alone and the combination of three markers for the diagnosis of ovarian cancer

**Figure 2:**
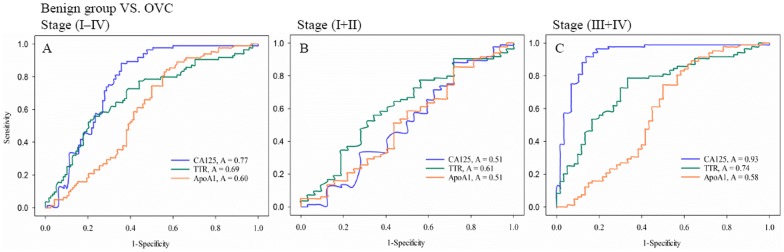
ROC (receiver operator characteristic) discriminating ovarian cancer patients from benign ovarian disease patients. The curves shown were obtained by processing quantified raw data by SigmaPlot 12.0 version software and the sensitivity/specificity values were predicted from the area under the curves and the calculated data. ROC curves for CA125, transthyretin, and apolipoprotein A1 alone: (A) benign patients versus patients with ovarian cancer; (B) benign patients versus patients with stages I to II ovarian cancer; (C) benign patients versus patients with stages III to IV ovarian cancer.

Then we compared the sensitivity and specificity between each marker alone and the three markers in combination in order to diagnose ovarian cancer using receiver operating characteristic (ROC) analysis. In this study, we used cut-off values of 35 U/ml, 100 ng/ml, and 500 ng/ml for CA125, transthyretin, and apolipoprotein A1, respectively, for better diagnostic accuracy for the samples tested here. By using these cut-off values, we were able to minimize the rates of false-positive and false-negative findings in the differentiation of benign patients from subjects with ovarian cancer. The sensitivities and specificities for discriminating between ovarian cancer and benign disease are shown in Figure 2. The sensitivity and specificity of individual markers with CA125, transthyretin, and apolipoprotein A1 were 77.4% and 70.8%, 69.7% and 63.6%, and 60.2% and 56.9%, respectively (Fig. 2A). The sensitivity and specificity of the individual markers for early-stage (stages I and II) were 51.6% and 51.2%, 61.2% and 59.2%, and 51.3% and 54.6%, respectively (Fig. 2B). And the sensitivity and specificity of the individual markers for late-stage (stages III and IV) were 93.4% and 84.3%, 74.9% and 68.2%, and 58.4% and 55.1%, respectively (Fig. 2C). By simple view of the curves, the discriminatory power in Fig. 2A is modest yet better for CA125, in Fig. 2B, very weak for all biomarkers with some preference of TTR, and in Fig. 2C, clearly strongest for CA125 followed by TTR and useless for ApoA1.

**Figure 3:**
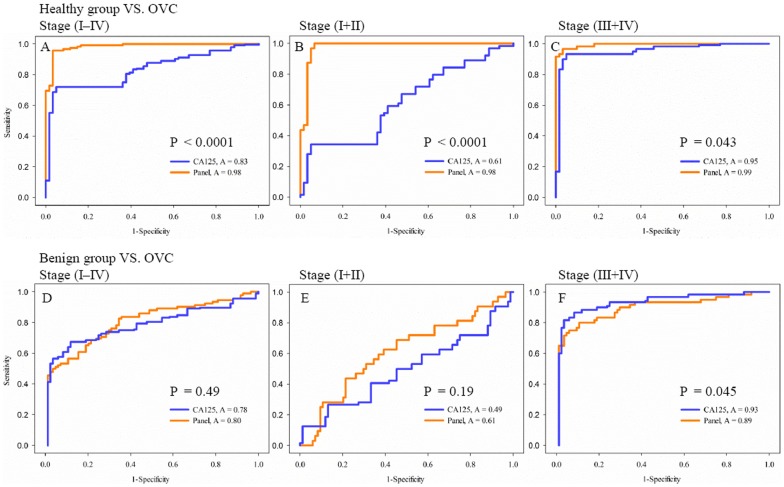
ROC discriminating ovarian cancer patients from healthy controls or benign ovarian disease patients using the three-biomarker panel. The curves shown were obtained by processing quantified raw data by SAS 9.1 version software and the sensitivity/specificity values were predicted from the area under the curves and the calculated data. ROC curves for CA125 alone and the three-biomarker panel: (A) healthy controls versus patients with ovarian cancer. The overall difference in AUCs between the three-biomarker panel and CA125 alone was statistically significant (P<0.0001); (B) healthy controls versus patients with stages I to II ovarian cancer (P<0.0001); (C) healthy controls versus patients with stages III to IV ovarian cancer (P = 0.043); (D) benign patients versus patients with ovarian cancer. (P = 0.49); (E) benign patients versus patients with stages I to II ovarian cancer (P = 0.19); (F) benign patients versus patients with stages III to IV ovarian cancer (P = 0.045).

When CA125 was combined with the biomarkers (transthyretin and apolipoprotein A1), the overall sensitivity and specificity for discriminating between ovarian cancer and healthy individuals were significantly improved in the ROC curve (Fig. 3A–C). The three biomarkers (CA125, TTR, and Apo-A1) significantly distinguished patients with early stage ovarian cancer from healthy individuals. At 95% specificity for all stages the sensitivity increased to 95.5% compared to 67% for CA125 alone. For stage I+II increased the sensitivity to 93.9% from 30% for CA125 alone. For stage III+IV were the corresponding values respectively 96.5% and 91.6%, suggesting that the three-biomarker panel classified early-stage cancers with 94% sensitivity at 95% specificity, which was significantly higher than CA125 alone. The overall sensitivity and specificity for discriminating between ovarian cancer and benign ovarian disease were slightly improved in the stage I+II patient group only, which showed 61.6% and 58% sensitivity and specificity, respectively (Fig. 3D–F). In the figure, it is evident that the difference between the CA125 and the panel for healthy vs. total and stage I+II ovarian cancer is highly significant (P<0.0001) but negligible for healthy vs. stages III+IV cancer (P = 0.043); in contrast for the more important differentiation between benign vs. ovarian cancer, the difference between CA125 and the panel is missing for benign vs. total and stage I+II ovarian cancer and rather better for benign vs. stage III+IV ovarian cancer (P = 0.045). As the most important discrimination is generally postulated between a malignant group and its differentially relevant benign disease group, it becomes clear that the difference between CA125 and the panel is slightly better for benign vs stages III+IV.

**Figure 4:**
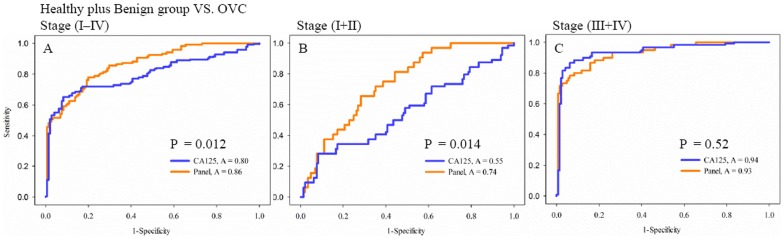
ROC discriminating ovarian cancer patients from healthy controls plus benign ovarian disease patients using the three-biomarker panel. ROC curves for CA125 alone and the three-biomarker panel: (A) healthy controls plus benign patients versus patients with ovarian cancer. (P = 0.012); (B) healthy controls plus benign patients versus patients with stages I to II ovarian cancer (P = 0.014); (C) healthy controls plus benign patients versus patients with stages III to IV ovarian cancer (P = 0.52).

The three biomarkers were sufficient for maximum separation between healthy plus benign group and stage I+II or all stages (I–IV) (Fig. 4). The combined groups of healthy and benign patients were tested by ROC analysis versus total and stage I+I or stage III+IV ovarian cancer groups by estimating a mixture between the Fig. 3A–C and 3D–F results. Indeed, the AUC testing for significant differences between CA125 and the three-biomarker panel shows no difference versus stages III+IV and clear but modest significant differences versus the total (P = 0.012) and stages I+II ovarian cancer (P = 0.014).

### Validation

**Figure 5:**
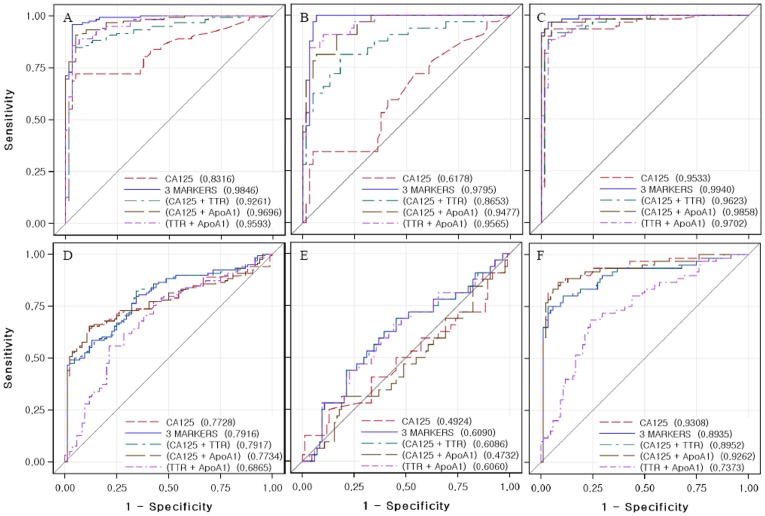
ROC discriminating ovarian cancer patients from healthy controls or benign ovarian disease patients using CA125, the two and three-biomarker panels. ROC curves for CA125 alone, the two and three-biomarker panels: (A) healthy controls versus patients with ovarian cancer; (B) healthy controls versus patients with stages I to II ovarian cancer; (C) healthy controls versus patients with stages III to IV ovarian cancer; (D) benign patients versus patients with ovarian cancer; (E) benign patients versus patients with stages I to II ovarian cancer; (F) benign patients versus patients with stages III to IV ovarian cancer.

To confirm whether this combination is the highest accuracy of multivariate classification algorithms, we used two-biomarker panels (CA125 plus transthyretin, CA125 plus apolipoprotein A1, and transthyretin plus apolipoprotein A1). The overall sensitivity and specificity of these two-biomarker panels were not improved for discriminating between ovarian cancer and healthy individuals as compared to the three-biomarker panel in the ROC curve (Fig. 5A–C), suggesting that the three-biomarker panel showed the highest accuracy, while the two-biomarker panels showed similar trends with the three-biomarker panel between a malignant group and its benign disease group (Fig. 5D–F). And the three-biomarker panel was sufficient for maximum separation between healthy plus benign group and stage I+II or all stages (I–IV) (Figure S1).

Starting with a population of ovarian cancer patients, stratified simple random sampling without replacement was used. For validation, 50% of these patients were to be used as a training set to establish the benefit of the combination of these markers for the diagnosis of ovarian cancer. The remainder of the patients served as the validation set for independently validating the usefulness of this biomarker. The training and validation sets were very similar with respect to patient characteristics. In the training set, the overall sensitivity and specificity of the three-biomarker panel showed the highest accuracy, while the two-biomarker panels showed less sensitive trends than the three-biomarker panel in the ROC curve (Figure S2, S3, S4). In the validation set, the three-biomarker panel showed the highest accuracy (Figure S5, S6, S7).

To see whether this combination gives the highest classification power in multivariate classification algorithms, we used four-biomarker panel (CA125 plus transthyretin plus apolipoprotein A1 plus hemoglobin). The overall sensitivity and specificity of these four-biomarker panel were not improved for discriminating between ovarian cancer and healthy individuals (and/or benign group) as compared to the three-biomarker panel in the ROC curve (Figure S8–S9), suggesting that the three-biomarker panel is sufficient for maximum separation between healthy (and/or benign group) and stage I+II or all stages (I–IV). Taken together, the three-biomarker panel could be a powerful biomarker for diagnosis of ovarian cancer.

## Discussion

Conventional ovarian cancer screening tools are ineffective for the general population [3]. The most studied marker for ovarian cancer, CA125, is a protein that is found at levels in most ovarian cancer cells that are elevated compared to normal cells and a potentially useful marker for diagnosis and prognosis after treatment of ovarian cancer, but CA125 is expressed in only 50–60% of patients with early-stage disease, and is also frequently elevated in women with benign ovarian diseases [20]–[22]. Due to the vulnerable points of CA125 as a biomarker of ovarian cancer [23], combining one or more other tumor markers with CA125 might improve the sensitivity and specificity of the diagnosis of ovarian cancers or the earlier detection of such cancers [9]. Thus, considerable efforts have been deployed to get a minimum Positive Predictive Value (PPV) of 10% and a specificity of greater than 99% as an effective ovarian cancer screening test [24], [25]. In 2009, a clinical test (OVA1) was approved by the FDA. The test was based on the estimation of the levels of five proteins (transthyretin, apolipoprotein A1, transferrin, beta-2 microglobulin, and cancer antigen 125) in blood, which was then combined into a single score, ranging from 0 to 10, using a unique algorithm (OvaCalc). While no published studies exist for OVA1 [26], it was reported that the OvaCalc algorithm performance showed 92.5% sensitivity, 43.0% specificity, 41.9% positive predictive value, and 92.9% negative predictive value. And among the 96 patients diagnosed with epithelial ovarian cancer, OvaCalc designated all but 1 as high risk. But it was not reported how many women with benign ovarian conditions were incorrectly categorized as at high risk for malignancy, but this number is presumed to be considerable. In addition to currently insufficient evidence (e.g. insufficient published evidence), the test is not approved as a screening for early-stage ovarian cancer and may lead to greater amounts of false-positive results as a screening tool [26]. Thus further studies are needed to improve the specificity and sensitivity of the combined biomarkers in both retrospective and prospective clinical trials as a screening tool. Some of the test results have been published [23]. However, to date no screening test has achieved adequate performance characteristics to be used as a valuable tool for the detection of early stage ovarian cancer. In this study, we evaluated a new combination of three known biomarkers of ovarian cancer, CA125, transthyretin, and apolipoprotein A1, in an attempt to improve the sensitivity of CA125, showing that transthyretin and apolipoprotein A1 were increased the sensitivity and specificity of the CA125 in early stage ovarian cancer. While transthyretin and apolipoprotein A1 have been used several times as potential biomarkers of ovarian cancer [23], [27], the three-biomarker panel was newly evaluated using our Korean population. Moreover, this study effectively presented the validation of the use of a multiplex liquid assay system for the simultaneous detection of several biomarkers for the diagnosis of ovarian cancer. The cutoff 35 U/ml for CA125 we used is generally accepted as normal [28].

Transthyretin has been used as a biomarker for malnutritional status and inflammation, acute and chronic diseases, but post-translational modified forms have also been reported as part of a biomarker panel for early detection of ovarian cancer [29]–[31]. The serum level of full-length transthyretin was down-regulated among patients with later stage ovarian cancer relative to that in healthy controls and patients with colorectal, breast, or prostate cancer. It was identified that corresponding to the peak at m/z 12.8 kD, a truncated form of transthyretin showed a lack of the N-terminal ten amino acids. In addition to mutations on protein level, TTR exists in different isoforms [32]. Recently, a truncated variant of transthyretin together with apolipoprotein A1 and a connective tissue activating protein III were described as an efficient panel of new biomarkers for detecting early stage epithelial ovarian cancer in women [23].

Apolipoprotein A1 is the major protein component of high density lipoprotein (HDL) in plasma [33]. It was shown that Apolipoprotein A1 concentration in blood is reduced in different types of cancer [34]. Apolipoprotein A1 has been identified as a potential biomarker of ovarian cancer, colorectal cancer, chronic obstructive pulmonary disease and pancreatic cancer [29], [35], [36]. However, controversial observations were also reported including up-regulation of Apolipoprotein A1 in a variety of malignant tumors of ovarian, liver, breast [37]. Recently, apolipoprotein A1 was shown to enhance the sensitivity of CA125 for detecting early stage epithelial ovarian cancer and suggested a promising therapeutic agent for the treatment of ovarian cancer [23], [38].

However, when applied individually, the markers studied here did not surpass CA125 in their sensitivities and specificities in the diagnosis of ovarian cancer. Combining individual markers has been attempted by other researchers as one strategy to enhance the overall ovarian cancer detection rate [23], [39]–[41]. Here, we applied the combination of the two serum markers with CA125, and compared the sensitivities and specificities between the three-marker panel and each marker alone. Results from ROC curve analysis show that combining three biomarkers had a much improved sensitivity over that of each biomarker alone. The three-biomarker panel classified early-stage cancers with 93.9% sensitivity and late-stage cancers with 96.5% sensitivity at 95% specificity. We also added hemoglobin, one of our serum biomarkers published recently [42], into the panel to confirm whether this combination gives the highest classification power. But, the four-biomarker panel (CA125 plus transthyretin plus apolipoprotein A1 plus hemoglobin) did not improve the overall sensitivity and specificity for discriminating between ovarian cancer and healthy individuals (and/or benign group) as compared to the three-biomarker panel in the ROC curve. Thus, regardless of hemoglobin, the three-biomarker panel was sufficient for maximum separation between noncancer (healthy plus benign group) and stage I+II or all stages (I–IV) of disease. The sensitivity and specificity of this panel for stage I+II are comparable to results with a four-biomarker panel selected from 96 candidate antigens measured by immunoassays with multiplex techniques [43]. Their panel of biomarkers correctly classified 67% of benign lesions as noncancer. Another study demonstrated the clinical utility of a CA125/HE4 combined test for the discrimination of benign and malignant ovarian masses with 76.4% sensitivity at 95% specificity [44]. The high specificity and corresponding increases in sensitivity for the three-biomarker panel has merit in ovarian cancer screening trials. It was, however, reported that for the general population, the PPV of a 6-marker panel (leptin, prolactin, OPN, IGF-II, MIF, CA-125) measured by a multiplex bead-based immunoassay system would be 6.5%, indicating that 14 out of 15 women with a positive test result would experience false-positive test results [45].

There are several limitations that need to be addressed regarding the present study. First, a clearer description of population such as age and racial distribution, nutritional status, presence of peritoneal carcinomatosis and ascites, presence of infection disease, exclusion of autoimmune disease or another malignancies (such as metastatic ovarian cancers from gastrointestinal system) could be very important for the evaluation of biomarker levels [46]–[49]. Second, natural biological variation of certain markers and individual biological variation over time should also be considered as the associated variability could induce a number of assay measurement error by false-positive results [50], [51]. For example, the inherent intra-individual biological variation in CA125 was greater in premenopausal than in postmenopausal females [52]. And even in a multi-center case-control study, there may be biomarker concentration differences. A 4 marker-panel (apolipoprotein A1 + transthyretin + inter-α-trypsin inhibitor IV + CA125), for example, exhibited concentration differences between biomarker discovery set and independent validation set [17]. It has been accepted that there may be demographic and epidemiological differences, and sample processing protocols differences between hospitals, leading to different results. Thus, a model for estimating analytical and biological components of variation of markers is needed. Then, the careful evaluation of screening performances in appropriate sample cohorts would be required to further improve the specificity and sensitivity of the combined biomarkers in both retrospective and prospective clinical trials and lead to increased survival [8], [9], [53]. Third, a multimarker bead-based system has several benefits for the immunoassay using clinical samples compared with the conventional enzyme-linked immunosorbent assay techniques and proteomic-based analyses [54]. However, there are some difficulties inherent to the set up for multiplexing. A good pair of capture antibody and detection antibody should be determined, and cross-reactivity among different antibodies for multiplexing should be avoided using application-specific antibody validation [55]. Antibody cross-reactivity may produce a large background signal, thereby decreasing assay sensitivity. Theoretically, suspension assays are more vulnerable to cross-reactivity because cross-linking may occur as beads circulate in fluid, a factor that may limit the ability to multiplex [56]. Several commercially available Luminex multiplex panels have been compared with conventional commercial ELISAs for measurement of biomarkers in human plasma that are associated with obesity and inflammation [57], showing that significantly improved and faster validation methods would be available for ovarian cancer research. In addition to adjusting cross reactivity, assay diluents, optimal temperatures, incubation times, concentrations of reagents, and analytical validation of assay performance must be configured during multiplex assay development [58].

The present study showed the significant improvement of sensitivity for the diagnosis of ovarian cancer when using a combination of three serum biomarkers, including CA125, transthyretin, and apolipoprotein A1, using a multiplex liquid assay system. Further studies are going to be extended to a large number of ovarian cancer patients in early and late stages, as well as patients with benign ovarian diseases, in order to confirm the validity of the combination of these markers for the diagnosis at an early stage of ovarian cancer.

## Supporting Information

Figure S1 ROC discriminating ovarian cancer patients from healthy controls plus benign ovarian disease patients using CA125, three kinds of two-biomarker panels and a three-biomarker panel.
(TIF)

Figure S2 ROC curves for training set of healthy controls versus patients with ovarian cancer.
(TIF)

Figure S3 ROC curves for training set of benign patients versus patients with ovarian cancer.
(TIF)

Figure S4 ROC curves for training set of healthy controls plus benign patients versus patients with ovarian cancer.
(TIF)

Figure S5 ROC curves for validation set of healthy controls versus patients with ovarian cancer.
(TIF)

Figure S6 ROC curves for validation set of benign patients versus patients with ovarian cancer.
(TIF)

Figure S7 ROC curves for validation set of healthy controls plus benign patients versus patients with ovarian cancer.
(TIF)

Figure S8 ROC discriminating ovarian cancer patients from healthy controls using the four-biomarker panel (CA125 plus transthyretin plus apolipoprotein A1 plus hemoglobin). ROC curves for CA125 alone and the four-biomarker panel.
(TIF)

Figure S9 ROC discriminating ovarian cancer patients from benign ovarian disease patients using the four-biomarker panel (CA125 plus transthyretin plus apolipoprotein A1 plus hemoglobin). ROC curves for CA125 alone and the four-biomarker panel.
(TIF)

File S1 Reporting Recommendations for Tumor Marker Prognostic Studies (REMARK) compliance for multiplexed bead-based immunoassays for the detection of early stage ovarian cancer using a combination of serum biomarkers.
(DOC)
