# Supplementary figures and images for: Development of Multiplexed Bead-Based Immunoassays for the Detection of Early Stage Ovarian Cancer Using a Combination of Serum Biomarkers
Source: PLoS One. 2012 Sep 10;7(9):e44960. doi: 10.1371/journal.pone.0044960 (PMC3438175; doi:10.1371/journal.pone.0044960)

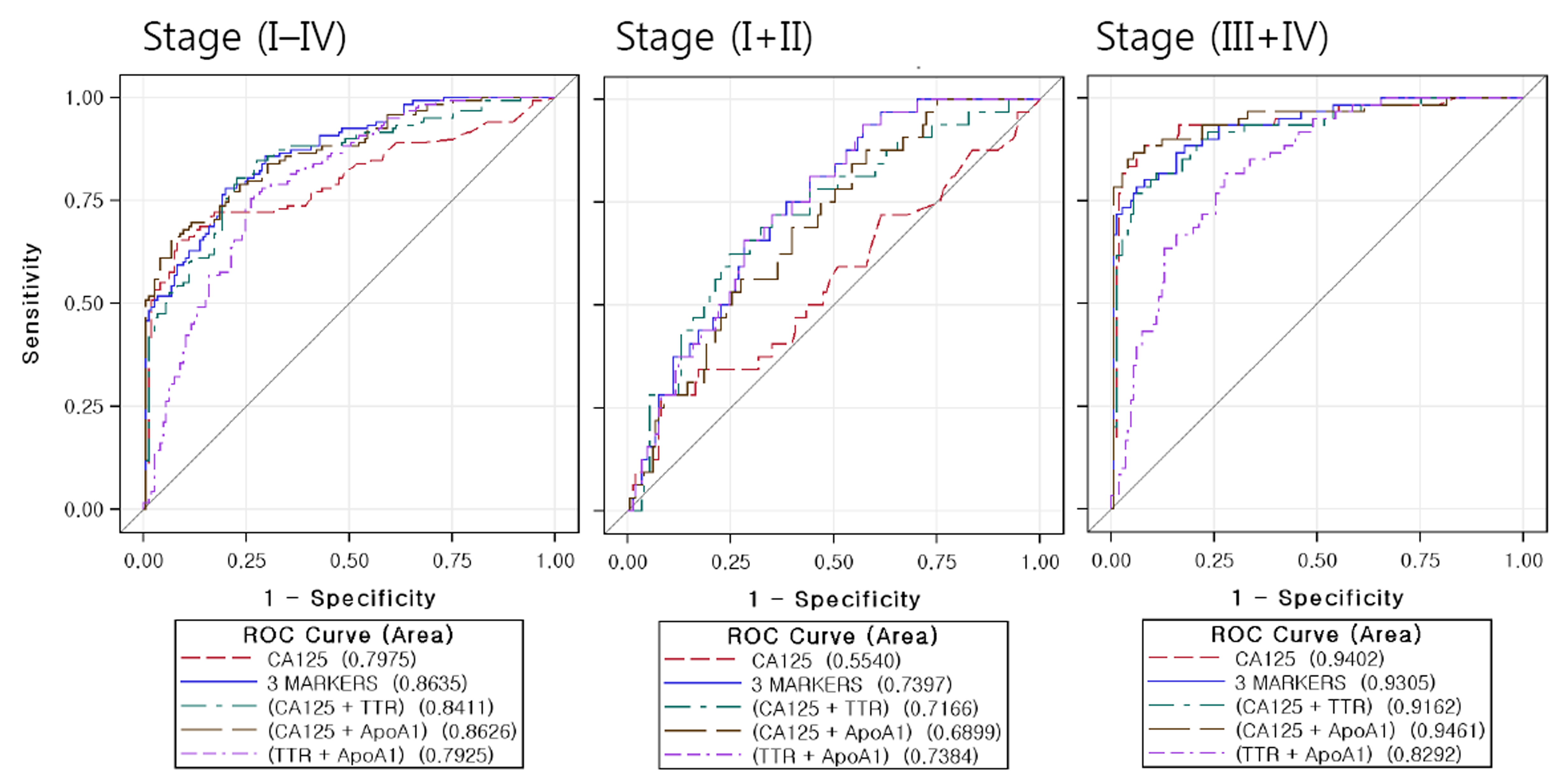

Supplement: Figure S1 — ROC discriminating ovarian cancer patients from healthy controls plus benign ovarian disease patients using CA125, three kinds of two-biomarker panels and a three-biomarker panel. (TIF) [file pone.0044960.s001.tif]

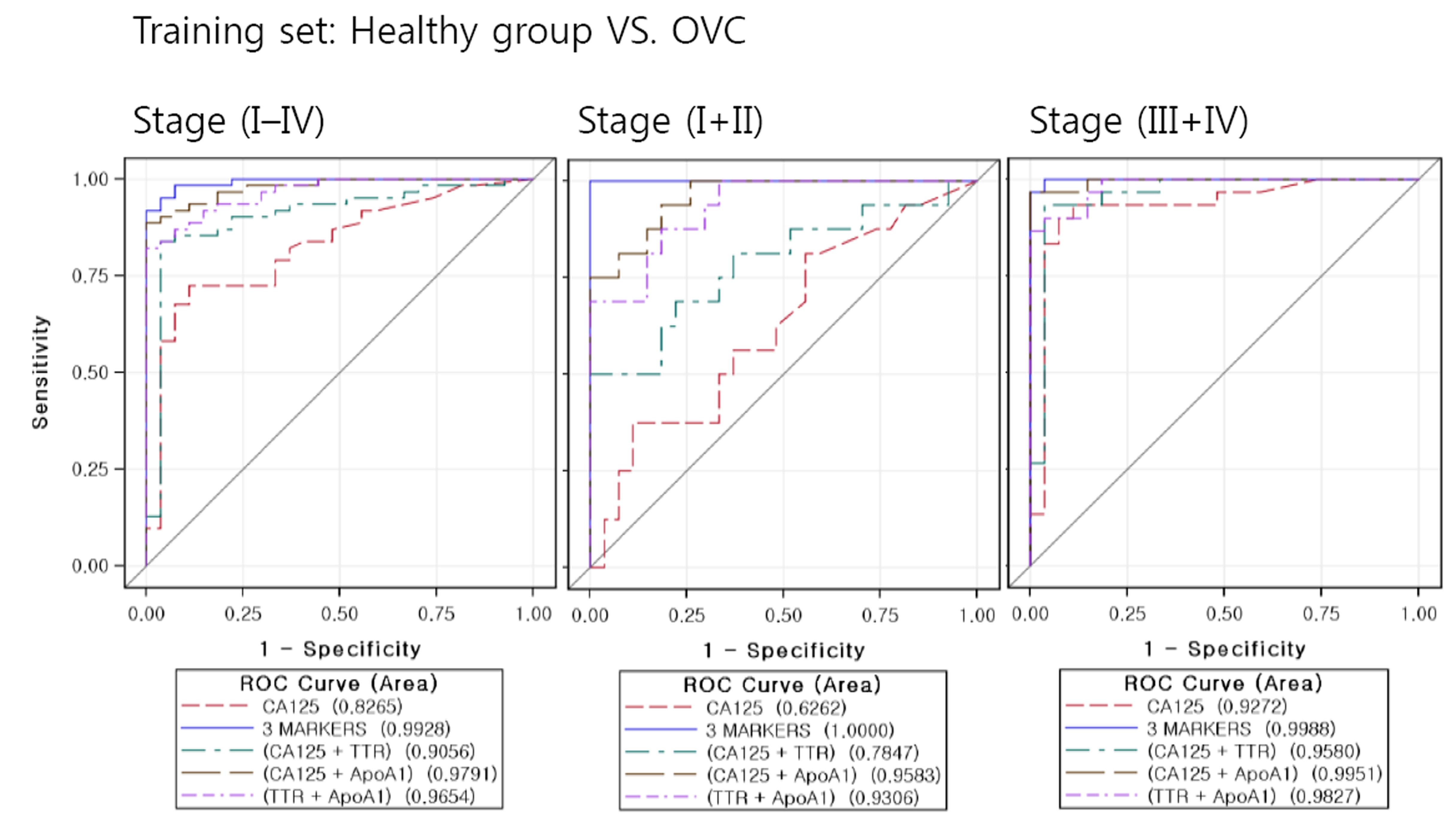

Supplement: Figure S2 — ROC curves for training set of healthy controls versus patients with ovarian cancer. (TIF) [file pone.0044960.s002.tif]

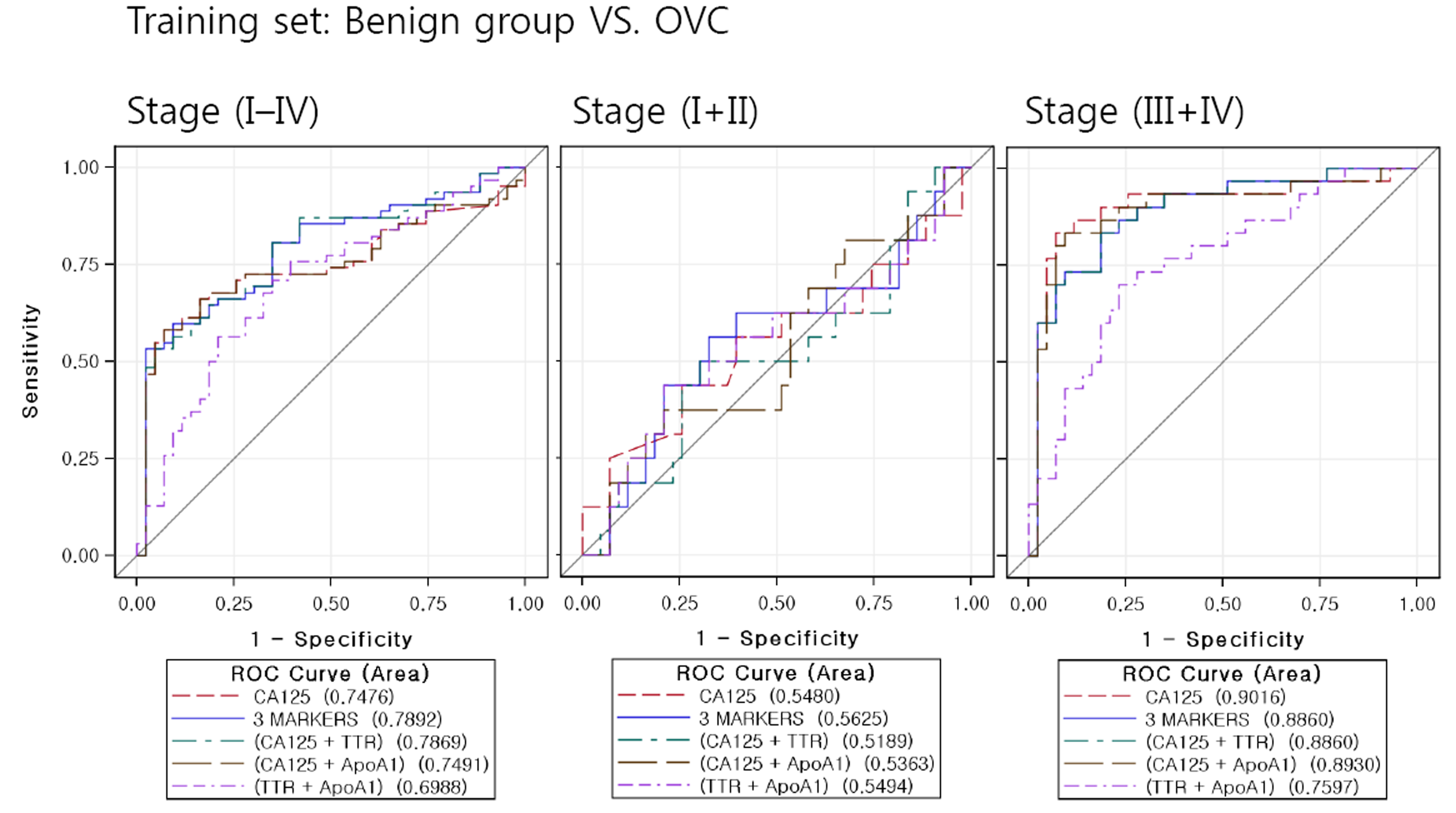

Supplement: Figure S3 — ROC curves for training set of benign patients versus patients with ovarian cancer. (TIF) [file pone.0044960.s003.tif]

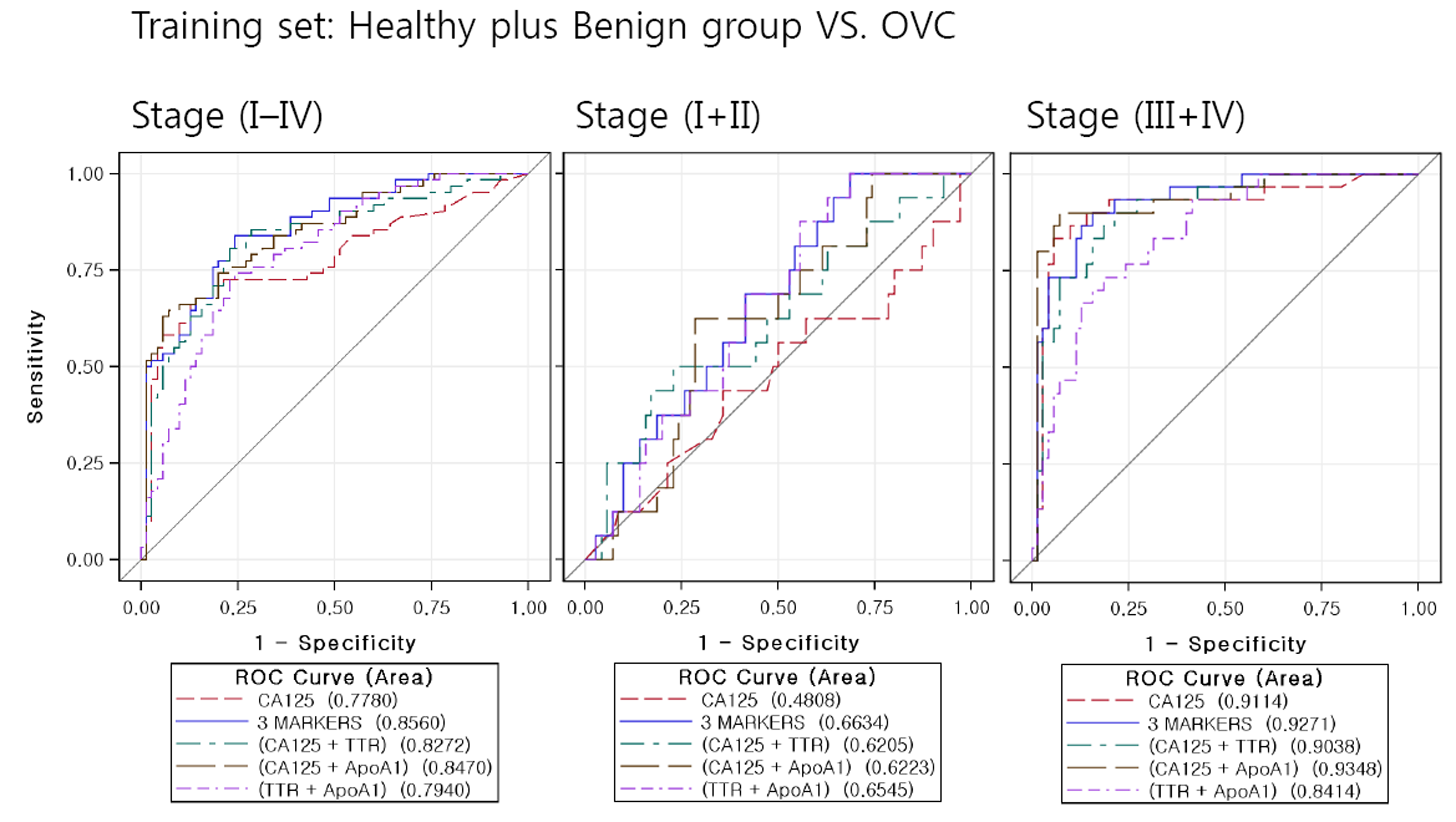

Supplement: Figure S4 — ROC curves for training set of healthy controls plus benign patients versus patients with ovarian cancer. (TIF) [file pone.0044960.s004.tif]

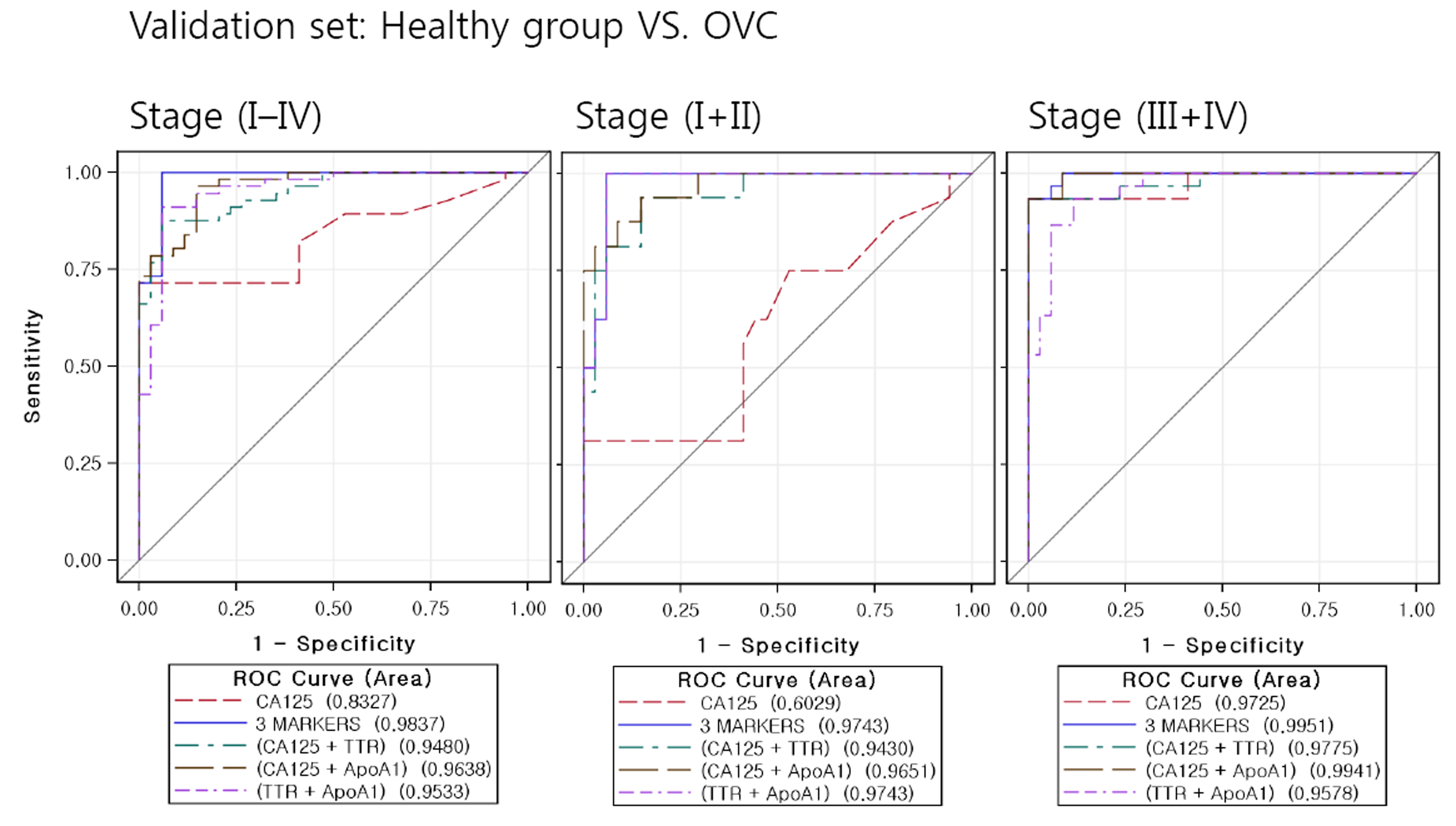

Supplement: Figure S5 — ROC curves for validation set of healthy controls versus patients with ovarian cancer. (TIF) [file pone.0044960.s005.tif]

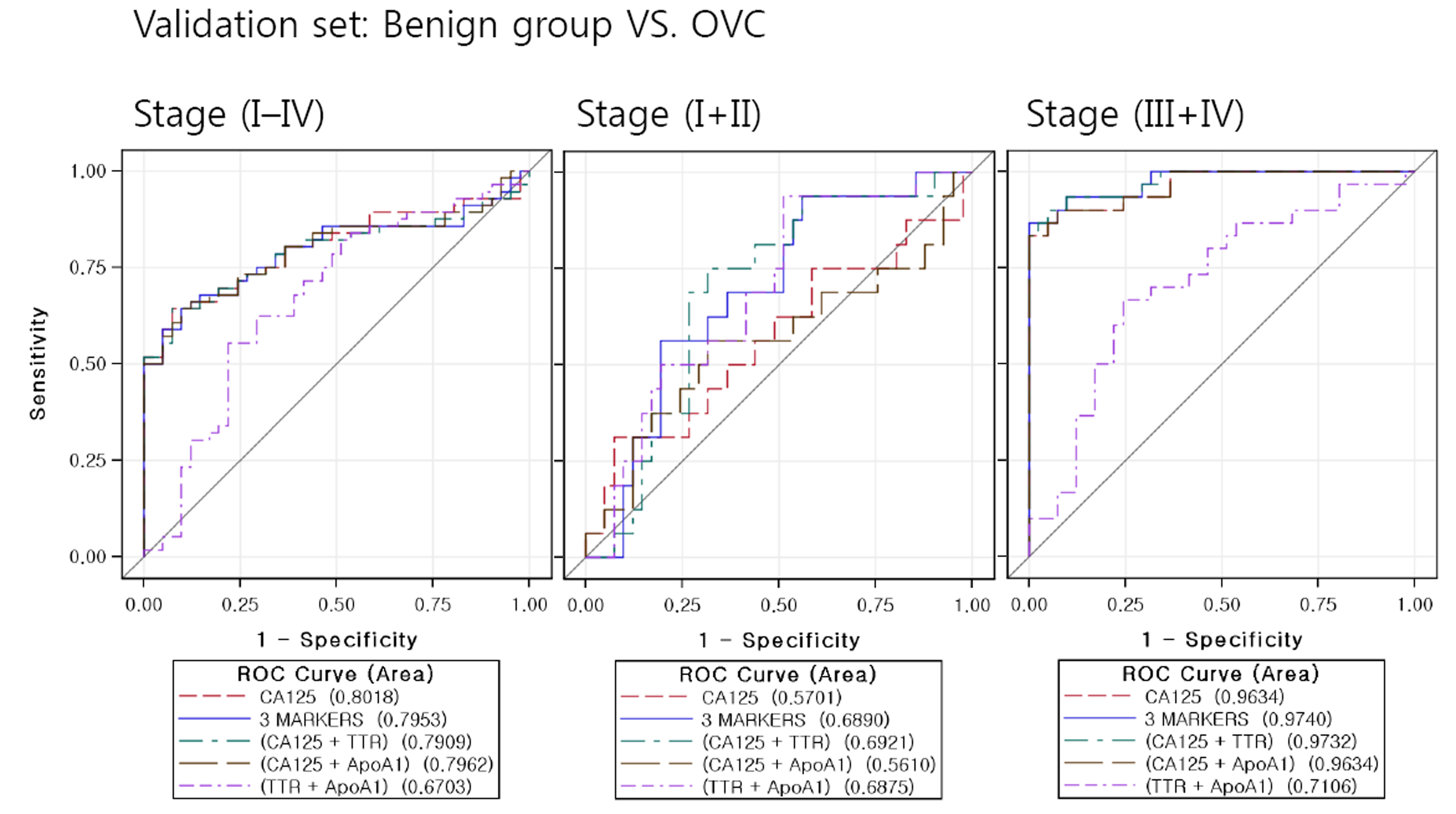

Supplement: Figure S6 — ROC curves for validation set of benign patients versus patients with ovarian cancer. (TIF) [file pone.0044960.s006.tif]

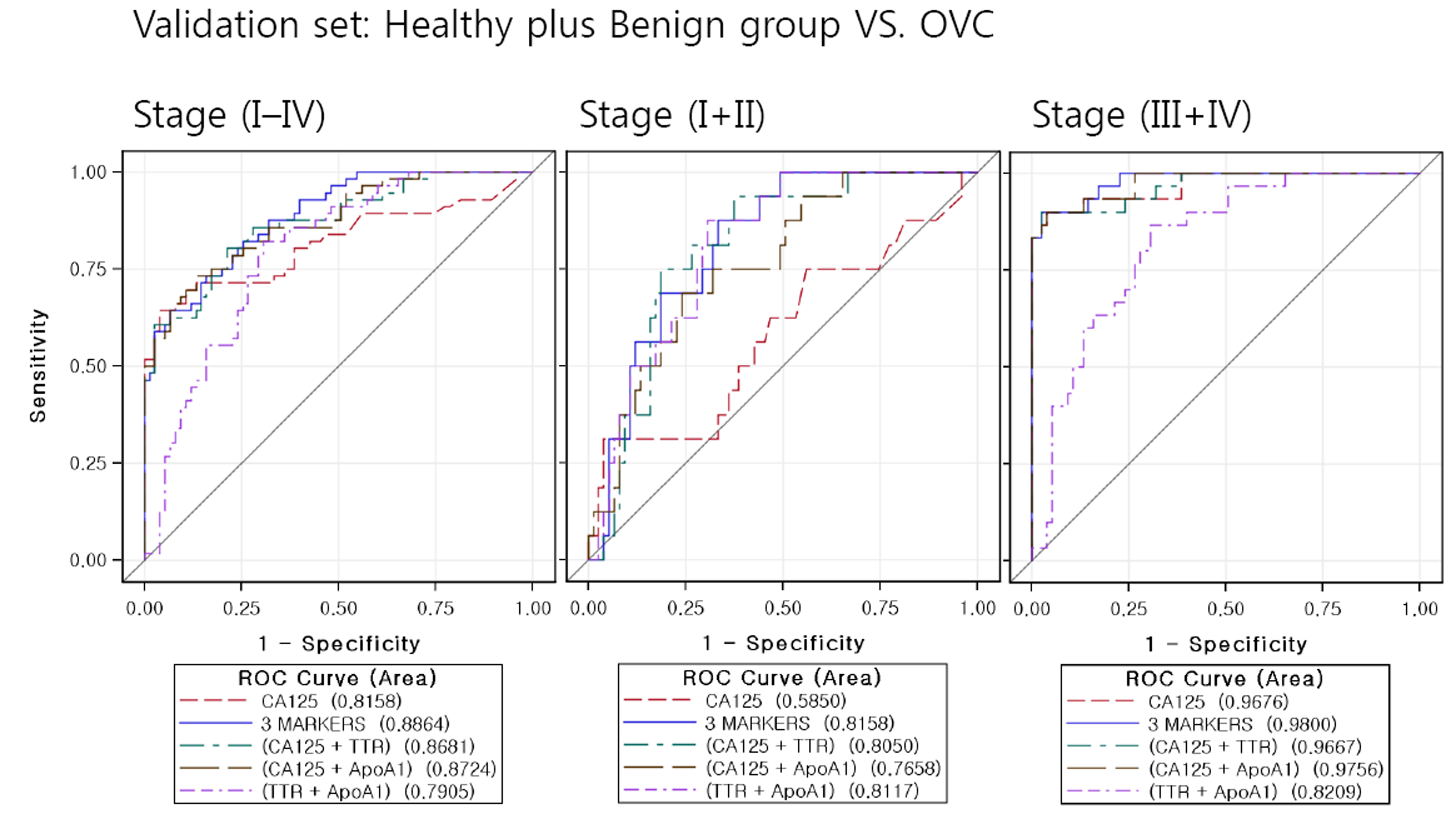

Supplement: Figure S7 — ROC curves for validation set of healthy controls plus benign patients versus patients with ovarian cancer. (TIF) [file pone.0044960.s007.tif]

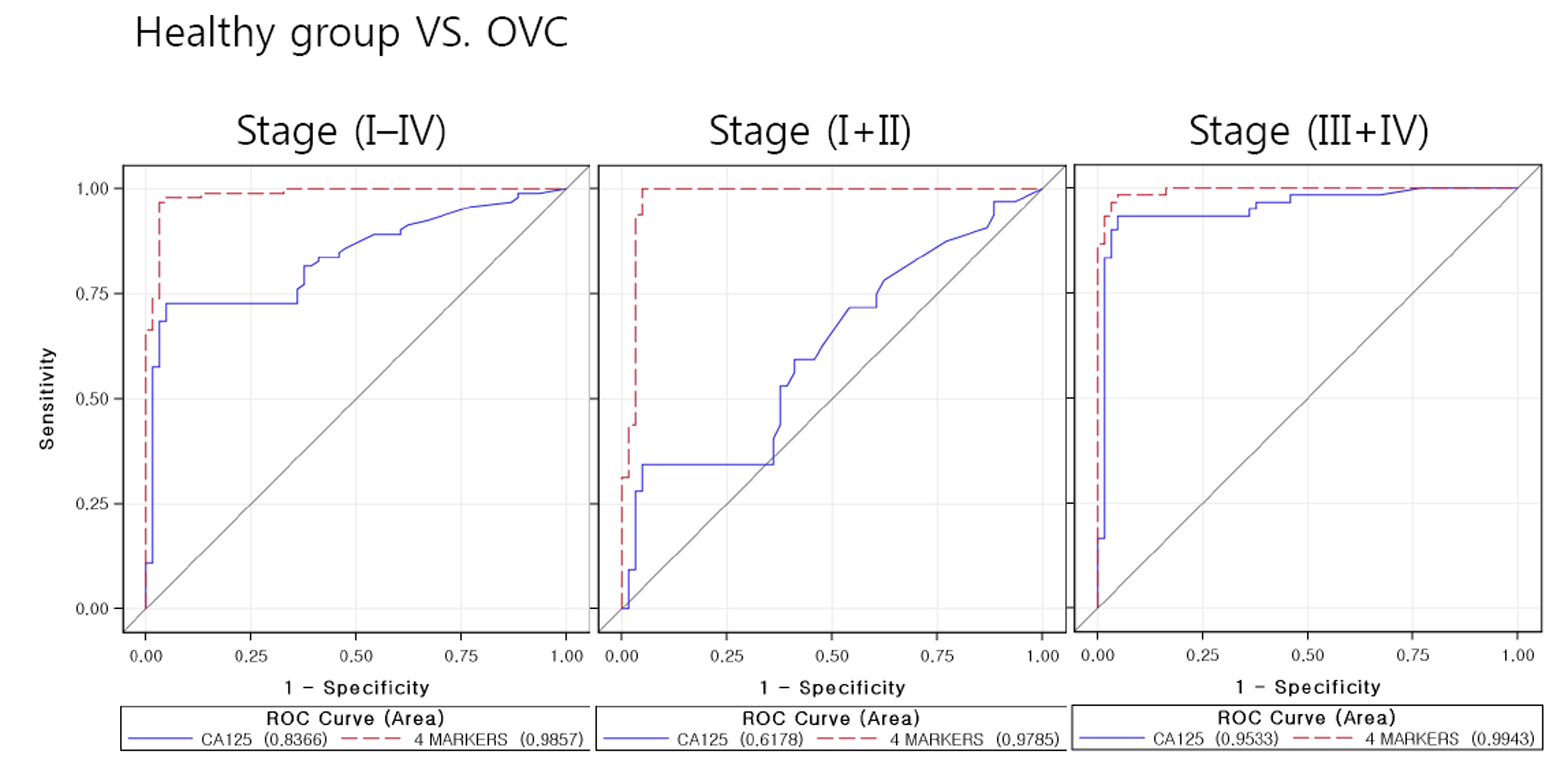

Supplement: Figure S8 — ROC discriminating ovarian cancer patients from healthy controls using the four-biomarker panel (CA125 plus transthyretin plus apolipoprotein A1 plus hemoglobin). ROC curves for CA125 alone and the four-biomarker panel. (TIF) [file pone.0044960.s008.tif]

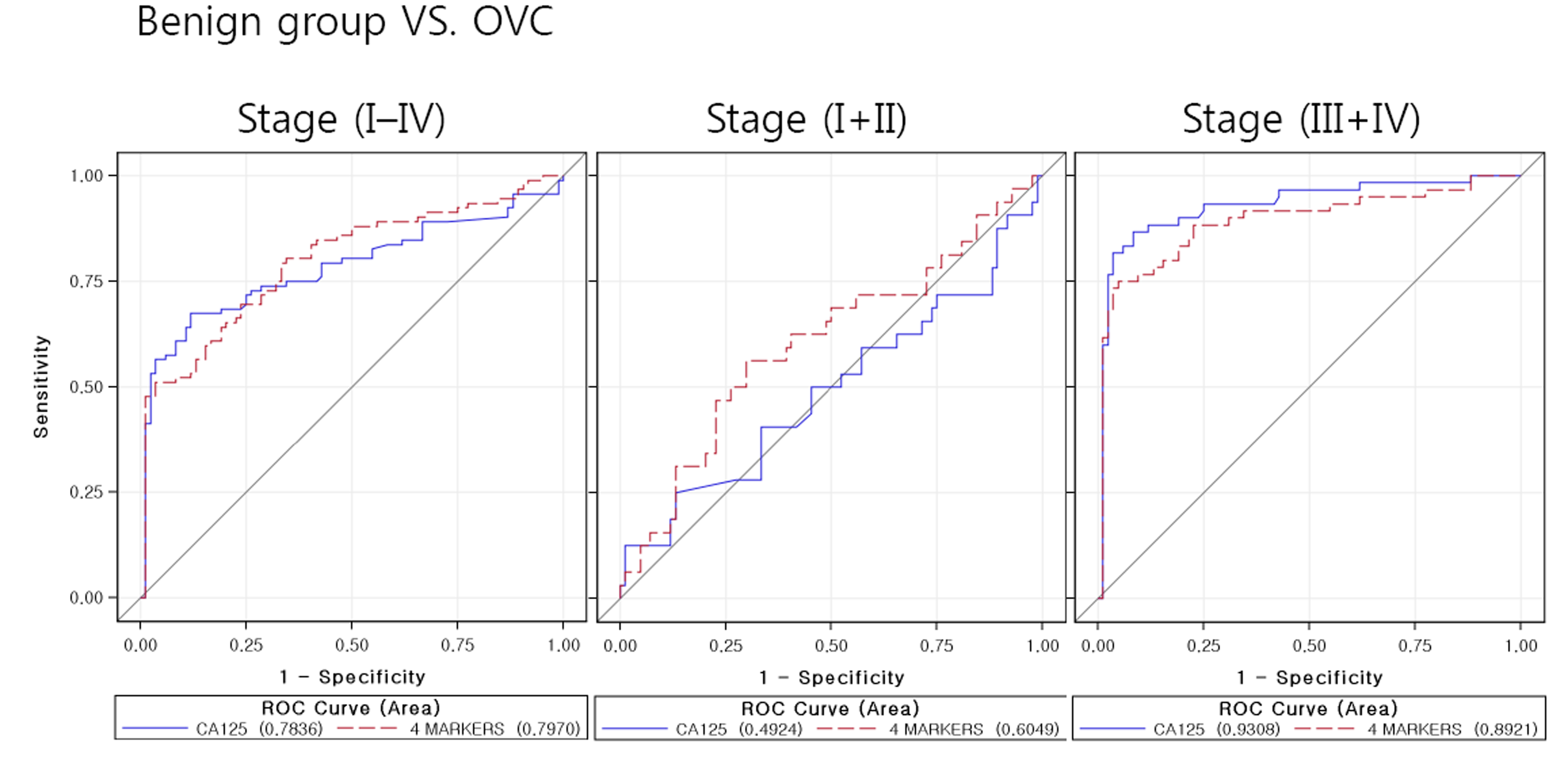

Supplement: Figure S9 — ROC discriminating ovarian cancer patients from benign ovarian disease patients using the four-biomarker panel (CA125 plus transthyretin plus apolipoprotein A1 plus hemoglobin). ROC curves for CA125 alone and the four-biomarker panel. (TIF) [file pone.0044960.s009.tif]
