# Supplementary material for: Development of Multiplexed Bead-Based Immunoassays for the Detection of Early Stage Ovarian Cancer Using a Combination of Serum Biomarkers
Source: PLoS One. 2012 Sep 10;7(9):e44960. doi: 10.1371/journal.pone.0044960 (PMC3438175; doi:10.1371/journal.pone.0044960)
Supplement: File S1 — Reporting Recommendations for Tumor Marker Prognostic Studies (REMARK) compliance for multiplexed bead-based immunoassays for the detection of early stage ovarian cancer using a combination of serum biomarkers. (DOC) [file pone.0044960.s010.doc]

**REMARK compliance for multiplexed bead-based immunoassays for the detection of early stage ovarian cancer using a combination of serum biomarkers**

**INTRODUCTION**

1. Marker examined:

CA125, apolipoprotein A1, transthyretin

Study objective:

To evaluate multiplexed bead-based immunoassay of multiple ovarian cancer-associated biomarkers such as transthyretin and apolipoprotein A1, together with CA125, to improve the identification and evaluation of prognosis of ovarian cancer.

Hypothesis:

A Luminex bead-based system is capable of measuring up to 100 analytes simultaneously in a small sample volume (less than 50 μl), indicating multivariate methods that use a panel of biomarkers to predict specific clinical end points of interest. Thus, the assay of three serum biomarkers of ovarian cancer, CA125, transthyretin, and apolipoprotein A1, using a multiplex bead-based immunoassay system, would evaluate the combined effect of the three biomarkers for the diagnosis of ovarian cancer compared with those of the individual markers alone.

**MATERIALS AND METHODS**

**Patients**

2. Source:

All patients were enrolled at St. Mary’s Hospital of Catholic Medical School during the period from January 2001 to July 2007, according to the procedures approved by the Institutional Review Board of The Catholic University of Korea.

**Stage:** Table 1 describes the characteristics of the study patients. Patients were stage I and II (34.8%), and stages III and IV (65.1%).

**Inclusion criteria:** Selected ovarian cancer patients with sufficient clinical data before surgery and follow up data after surgery.

**Exclusion criteria:** Ovarian cancer patients who received chemo or radiation therapy prior to serum collection

3. Treatment received for ovarian cancer patients: No applicable.

**Specimen characteristics**

4. Patient serum samples were collected before surgery, and then incubated for 30 min at room temperature, followed by centrifugation at 3,000 rpm for separation. The serum was stored at -70°C until used in experiments; frequent freezing and thawing were avoided.

**Assay methods**

5. Three different kinds of microspheres (1x10^6^ microspheres for each antibody, Biosource, Camarillo, CA) were prepared in each tube, and were then resuspended well by vortexing and sonication, followed by centrifugation for 2 min at 8,000 rpm. Supernatants were discarded, and the pellets were saved and washed once with 100 μl saline. Eighty μl of 100 mM monobasic sodium phosphate (pH6.2, Sigma-Aldrich, St. Louis, MO), 10 μl of 50 mM Sulfo-NHS (Pierce Biotechnology, Rockford, IL) and 10 μl of 50 mM EDC (Pierce Biotechnology) were added, and the solution was then incubated for 20 min at room temperature. After centrifugation (8,000 rpm, 2 min), the pellets were saved and washed twice with 250 μl of 50 mM MES (pH 5.0, Sigma-Aldrich). After the removal of the supernatant, 500 μl of MES was added to each tube including different microspheres. Following the addition of 0.5 μg of each antibody [anti-CA125 (Fitzgerald Industries International, Inc., Concord, MA), anti-transthyretin (Abcam), and anti-apolipoprotein A1 (Fizgerald Industries International, Inc.)] in each tube, the tubes were incubated for 2 h on a shaker, which was protected from the light. After the incubation, antibody-bound microspheres were pelleted by centrifugation for 2 min at 8,000 rpm, and 500 μl of 1% BSA buffer was then added. After additional incubation for 30 min at room temperature, the microspheres were washed twice with 1% BSA buffer and then stored at 4°C under protection from light.

For labeling biotins on the secondary antibodies, a biotin labeling kit (Alpha Diagnostics International Inc., San Antonio, TX) was used according to the protocol of the manufacturer. Briefly, biotin was added at a ratio of 1:10 (biotin:antibody). After incubation for 1 h at room temperature under protection from light, dialysis was performed with phosphate-buffered saline (PBS).

The serum from healthy individuals and ovarian cancer patients were diluted to 1:100 in a buffer including 1% BSA (Sigma-Aldrich) and 0.05% Tween 20 (Sigma-Adrich). Fifty μl of each diluted serum were placed on a 1.2 μm filter plate (96 well), to which 2,500 of each antibody-bound microsphere were added in 50 μl. After incubation for 2 h at room temperature under protection from light, they were washed twice with PBS buffer including 0.05% Tween 20. One hundred μl of 0.4 μg of streptavidin-R-phycoerythrin (Sigma-Aldrich) was added to each well, and plates were then incubated for 30 min, followed by two washes with PBS containing 0.05% Tween 20. The identification of antibody-bound microspheres and the screening of antigen-antibody-bound microspheres were carried out by using Luminex 100 (Luminex Corp, Houston, TX) according to the protocol of the manufacturer. Ranges of the concentrations of each antigen for standard curves were 10 – 250 U/ml for CA125, 0.1 – 100 μg/ml for transthyretin, and 0.5 – 50 ng/ml for apolipoprotein A1. The data were analyzed by the BeadView program (Upstate, Charlottesville, VA).

**Study design**

6. Case selection:

This study was based on analyses of serum collected from 118 patients with ovarian cancer, 84 with benign disease, and 61 healthy females that were retrospectively selected. The stages and grades of tumors from the ovarian cancer patients were assigned according to the guidelines provided by the International Federation of Gynecology and Obstetrics (FIGO), and the enrolled groups were then divided according to age. All patients were enrolled at St. Mary’s Hospital of Catholic Medical School during the period from January 2001 to July 2007, according to the procedures approved by the Institutional Review Board of The Catholic University of Korea.

7. Clinical end-points examined:

Patient serum samples that were eligible, were collected before surgery.

8. List of candidate variables: None

9. Rationale for sample size, including effect size to detect and power:

This study was designed as a retrospective study to evaluate multiplexed bead-based immunoassay of multiple ovarian cancer-associated biomarkers such as transthyretin and apolipoprotein A1, together with CA125 in 118 ovarian cancer patients vs. 61 healthy individuals and/or 84 patients with benign ovarian disease. The rationale for the total sample size of 263 derived as follows: Long (1997) suggests that sample sizes of less than 100 should be avoided and that 500 observations should be adequate for almost any situation (Long, J. Scott. 1997. Regression Models for Categorical and Limited Dependent Variables. Thousand Oaks, CA: SAGE Publications, Inc.). However, this leaves a relatively large gap between 100 and 500. If power analysis for determining the sample size for a research study gives a sample size of less than 100, researchers might want to increase it to at least 100, just to be safe. Similarly, Sudman (1976) suggests that a minimum of 100 elements is needed for each major group or subgroup in the sample and for each minor subgroup, a sample of 20 to 50 elements is necessary (Sudman, Seymour. 1976. Applied Sampling. New York: Academic Press.).

**Statistical analysis methods**

10. The primary statistical methods used were a receiver operating characteristic (ROC) curve and confidence intervals for evaluating diagnostic tests. We compared the performance of newly developed biomarker panel to a standard biomarker in the same sample population. Therefore, our study focuses on “paired” analyses. Additionally, non-parametric techniques were emphasized, as they are more widely applied in biomarker evaluation than parametric ROC analysis involving binormal assumptions. The diagnostic accuracy of a biomarker is most commonly measured by calculating its sensitivity and specificity. Since most diagnostic biomarkers provide results in the continuous scale (e.g. concentration of a tumor marker in serum measured in Unit/ml), the sensitivity and specificity of the test depends on the specific threshold selected. We used cut-off values of 35 U/ml, 100 ng/ml, and 500 ng/ml for CA125, transthyretin, and apolipoprotein A1, respectively, for better diagnostic accuracy for the samples tested here. Sensitivity and specificity were calculated for this threshold by constructing a 2 x 2 table using SAS (v9.1, SAS Institute, Cary, N.C., USA) (Shin, Sanghyuk. 2009. ROC analysis for the evaluation of continuous biomarkers: Existing tools and new features in SAS® 9.2. SAS Conference Proceedings.). With the predefined threshold, the confidence intervals for sensitivity and specificity were calculated using standard statistical methods for binomial data. Using BINOMIAL option along with the EXACT BINOMIAL statement, we produced confidence intervals using the normal approximation of the binomial distribution and the exact binomial distribution. ROC curves were generated using standard STATISTICAL GRAPHICS and simple LOGISTIC procedure statements. The area under the ROC curve (AUC) was used as a summary statistic representing the overall performance of the biomarker. The Mann-Whitney U-statistics was used for paired sample statistical techniques. With these tools, the ROCs of several biomarkers were compared by evaluating the difference of the AUCs which was asymptotically normal. We have no missing data for analyses.

11. Clarify how marker values were handled:

Not applicable as the marker values (the concentration of each marker in serum) were fixed in this study. For example, the cutoff 35 U/ml for CA125 we used is generally accepted as normal.

**RESULTS**

**Data**

12. Describe the flow of patients through the study:

The characteristics of patients and serum levels of ovarian cancer markers are shown in Table 1. Patient serum samples were collected before surgery. This study was not a staged analysis.

13. Report distribution of basic demographic characteristics:

The characteristics of patients and serum levels of ovarian cancer markers are shown in Table 1.

**Analysis and presentation**

14. The relation of the marker panel to the standard prognostic variable of ovarian cancer (CA 125) was analyzed; see Figure 3 and 4 that showed the results, including early detection and exact associated P-values.

15. Effect of each tumor marker on the evaluation of prognosis of ovarian cancer was analyzed in univariate analysis and presented as ROC (see Figure 2).

16. Not applicable, but effect of several tumor marker panels (two and three-biomarker panel) on the evaluation of prognosis of ovarian cancer was analyzed in multivariate analysis and presented as ROC (see Figure 3, 4, and 5).

17. The relation of the biomarker panels to the standard prognostic variable of ovarian cancer (CA 125) was analyzed; see Figure 3 and 4 that showed the results, including early detection and exact associated P-values.

18. The supplement Figures showed several additional analyses that the assay of three- biomarker panel of ovarian cancer was consistently evaluated the combined effect of the three biomarkers for the diagnosis of ovarian cancer compared with those of the individual markers alone.

**DISCUSSION**

19. Our results from the retrospective study supports our hypothesis that the new combination of transthyretin, and apolipoprotein A1 with CA125 improved both the sensitivity and the specificity of ovarian cancer diagnosis compared with those of individual biomarkers. Currently, a clinical test approved by the FDA in 2009 was based on the estimation of the levels of five proteins (transthyretin, apolipoprotein A1, transferrin, beta-2 microglobuliin, and cancer antigen 125) in blood. We discussed whether our biomarker combination is the highest accuracy of classification algorithms.

20. Our study provided a potential biomarker panel for predicting ovarian cancer. Future research will be to carry out a large prospective study on ovarian cancer patients.
